# A novel mutation in *TRIOBP* gene leading to congenital deafness in a Chinese family

**DOI:** 10.1186/s12881-020-01055-5

**Published:** 2020-06-01

**Authors:** Bingxin Zhou, Lili Yu, Yan Wang, Wenjing Shang, Yi Xie, Xiong Wang, Fengchan Han

**Affiliations:** 1 Key Laboratory for Genetic Hearing Disorders in Shandong, Binzhou Medical University, 346 Guanhai Road, Yantai, 264003 Shandong P. R. China; 2 Department of Biochemistry and Molecular Biology, Binzhou Medical University, 346 Guanhai Road, Yantai, 264003 Shandong P. R. China; 3 Reproductive Medicine Center, Affiliated Yantai Yuhuangding Hospital of Qingdao University, Yantai, 264000 China

**Keywords:** TRIOBP, DFNB28, Mutation, Hearing loss

## Abstract

**Background:**

The autosomal recessive non-syndromic deafness DFNB28 is characterized by prelingual sensorineural hearing loss. The disease is related with mutations in *TRIOBP* (Trio- and F-actin-Binding Protein) gene, which has three transcripts referred to as *TRIOBP-5*, *TRIOBP − 4* and *TRIOBP-1*. Among them, *TRIOBP-5/− 4* are expressed in the inner ears and crucial for maintaining the structure and function of the stereocilia.

**Methods:**

The proband is a 26-year-old Chinese female. She and her younger brother have being suffered from severe deafness since birth, whereas her parents, who are cousins, have normal communication ability. Hearing impairment of the two siblings was determined by pure tone audiometry. Whole Exome Sequencing (WES) was performed on the genomic DNA of the proband and Sanger sequencing was conducted on the DNA samples of the four family members.

**Results:**

Tests of pure tone hearing thresholds showed a severe to profound symmetric hearing loss for the proband and her younger brother. Moreover, a novel *TRIOBP* c.1342C > T (p.Arg448*) variant was identified by WES in the DNA sample of the proband and confirmed by Sanger sequencing in DNA of the family members.

**Conclusions:**

The *TRIOBP* c.1342C > T (p.Arg448*) variant is predicted to disrupt TRIOBP-5 and TRIOBP-4, which may lead to the congenital deafness. The results will broaden the spectrum of pathogenic variants in *TRIOBP* gene. The characteristics of deafness in the family imply that marriage between close relatives should be avoided.

## Background

As an autosomal recessive non-syndromic deafness, DFNB28 is characterized by pre-lingual sensorineural hearing loss. The disease is related with mutations in a gene named *TRIOBP* (Trio- and F-actin-Binding Protein) [1–5]. The *TRIOBP* gene in human and mouse encode three mRNA transcripts designated as *TRIOBP-5*, *TRIOBP-4* and *TRIOBP-1*, respectively. *TRIOBP-5* is the longest one covering both *TRIOBP-4* and *TRIOBP-1*. *TRIOBP-4* lies upstream of *TRIOBP-1* without overlapping with *TRIOBP-1*. In human, *TRIOBP-5, TRIOBP-4* and *TRIOBP-1* code 2365, 1144 and 652 amino acids, respectively [5]. The amino acid sequences of TRIOBP-5/− 4 contain two repeat motifs, which are referred to as R1 (amino acid residues 357–500) and R2 (amino acid residues 684–896). The R1 motif is the major actin-binding domain of TRIOBP-4, while the R2 motif’s binding with actin filaments is nonspecific [4, 6]. TRIOBP-5 or TRIOBP-1 has a pleckstrin homology domain (PH) and several coiled-coil domains (CC) [5]. In human and mouse, TRIOBP-5/− 4 are mainly expressed in the inner ears and retinas, and TRIOBP-1 plays an important role in embryonic development [2–4].

Many *TRIOBP* mutations associated with human DFNB28 have been identified [1, 2, 5, 7–10]. Though the variants cover the region from exon 4 to exon 23 (corresponding to amino acid residues from 52 to 2334), most of them are localized within exon 7 (exon 6 previously, corresponding to amino acid residues from 210 to1316), impairing the function of TRIOBP-5 and TRIOBP-4. Therefore, the exon 7 of *TRIOBP-5* is considered as the hotspot for variations [5]. The predicted protein changes due to variants in exon 7 of *TRIOBP-5* are summarized mainly as the followings: (p.Gln268fs), (p.Gln297*), (p.Arg347*), (p.Arg474*), (p.Arg523*), (p.Gln581*), (p.Gln740*), (p.Arg785fs), (p.Arg788*), (p.Arg861*), (p.Arg885fs), (p.Arg920*), (p.Ala998Thr), (p.Gly1019Arg), (p.P1030Lfs), (p.Ile1065Val), (p.Arg1068*), (p.Asp1069fs), (p.Arg1078fs), (p.Arg1078Cys), (p.Arg1117*), (p.Met1151Val), (p.Leu1154fs), (p.Glu1156*), (p.Arg1221Gln) and (p.Glu1314Asp). Among them, the variants of (p.Arg861*), (p.Arg920*), (p.Ile1065Val) and (p.Met1151Val) were found in the Chinese population [8, 9]. In this study, we further identified a novel *TRIOBP* c.1342C > T (p.Arg448*) variant in a Chinese family. The mutation is supposed to disrupt the isoforms of TRIOBP-5 and TRIOBP-4, leading to the congenital deafness of the family members.

### Clinical report

**Fig. 1:**
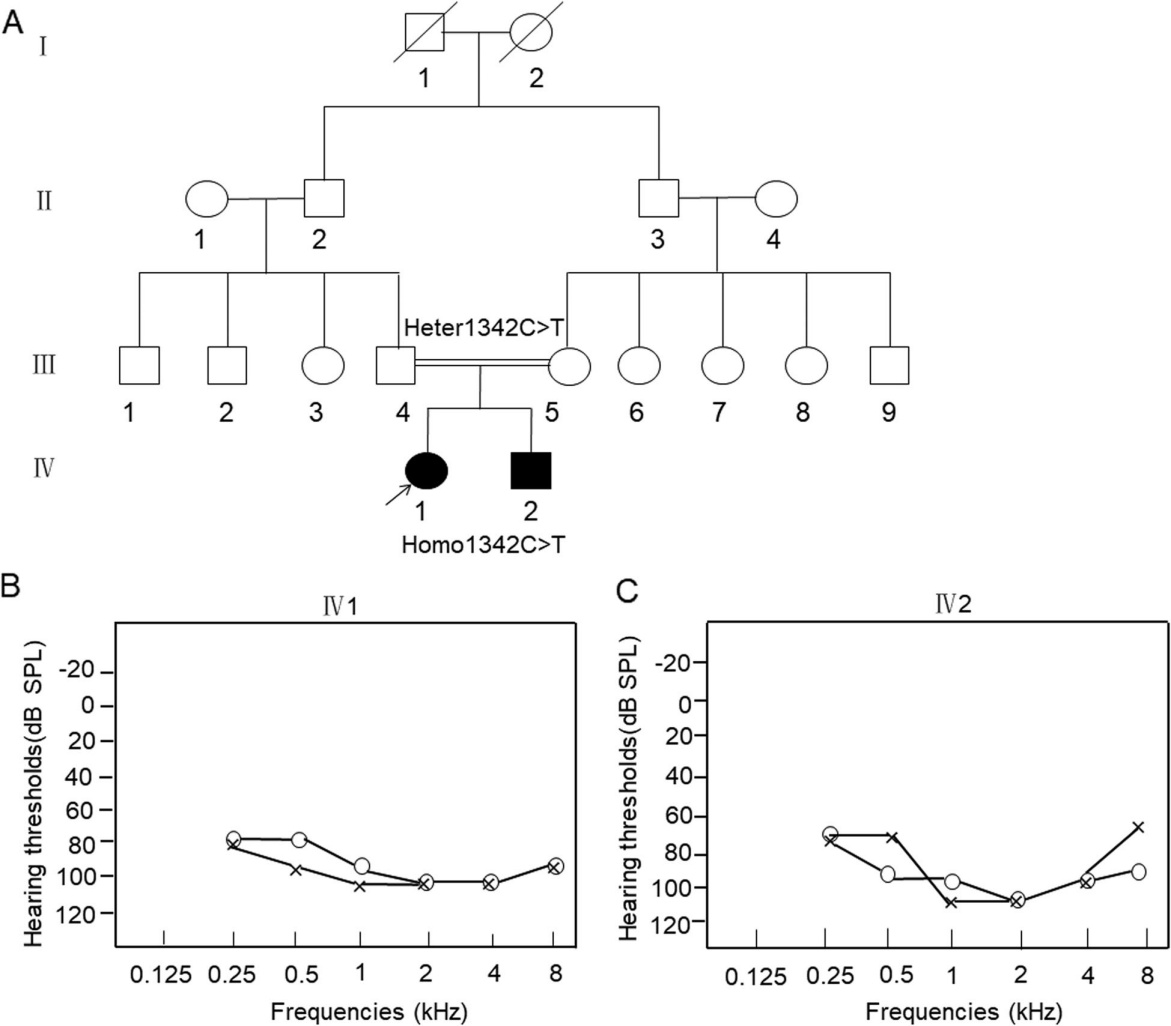
Identification of the individuals with hearing impairment. **a**. Pedigree analyses to identify the family members with hearing loss. The proband is indicated by an arrow. Genotypes are indicated as heterozygous 1342C > T (Heter1342C > T) for III34 and III45, and homozygous 1342C > T (Homo1342C > T) for (IV1 and IV2). **b**. Pure-tone audiograms to identify the hearing affected individuals. The individuals (IV1 and IV2) show severe to profound symmetric hearing impairment

A four-generation family is included in this study. All the family members have normal hearing with exception of two cases complaining of deafness in the fourth generation (Fig. 1). The proband (IV1), a 26-year-old female, has being suffered from severe deafness since birth. She was married and went to see a doctor in the Reproductive Medicine Center of the Yantai Yuhuangding Hospital for advice if she could have a healthy child. Her younger brother (the family member IV2) also suffered from serious hearing loss. Their parents are close relatives (cousins). No other symptoms were complained by the family members. This study was approved by the medical ethics committee of the Yantai Yuhuangding Hospital, Qingdao University. Informed consent was obtained from each of the subjects.

## Methods

### Test of brainstem evoked response audiometry

Clinical examination of the ear, nose and throat was performed on the two patients (IV1, IV2) and their parents (III4, III5) in the Department of Otolaryngology-HNS of Yuhuangding Hospital to exclude acquired causes of hearing impairment. Brainstem evoked response audiometry (BERA) was performed on the two patients (IV1, IV2) by using a computer-aided evoked potential system (Intelligent Hearing Systems, Miami, FL, USA). Auditory thresholds were obtained for stimuli of pure tone-bursts at 0.25, 0.5, 1, 2, 4, and 8 kHz in decibel sound pressure level (dB SPL).

### Identification of the gene variants

The peripheral blood samples were taken from the four subjects (IV1, IV2, III4, III5). Genomic DNA was isolated using the QIAGEN Universal DNA Purification Kit and diluted to a concentration of 50 ng/μl. The genome variants of the proband were identified by WES and confirmed by Sanger sequencing in Yin Feng Biotechnology Company (Jinan, China). The main procedures are described as followings: (1) DNA quality testing: After the DNA was extracted from the sample, the concentration was tested by Qubit® 3.0 Flurometer (Life Technologies, CA, USA), the purity was examined by a NanoPhotometer (IMPLEN, CA, USA) and the integrity was detected by 1% agarose gel electrophoresis; (2) Library construction: Library of small fragment preparation was conducted following the methods and procedures described in SureSelectXT Target Enrichment System (G7530–90000). The exons were then captured by a gene chip, eluted, recovered and enriched. (3) Testing the quality of the library. Agilent 2100 Bioanalyzer system was used to determine the insert sizes. Bio-Rad CFX 96 quantitative fluorescence PCR instrument and Bio-Rad KIT iQ SYBR GRN were used for Q-PCR to accurately determine the effective concentration of the library (> 10 nM). (4) Sequencing: double-end sequencing program (PE150) was performed on NovaSeq 6000 platform and sequence reads of 150 bp were received. (5) Raw data filtering and data comparison: reads containing primer/adaptor (> 5 bp) were removed and low-quality reads (N content in any sequencing read exceeds 5% of the read base number) were filtered; paired reads were removed when the number of low-quality bases(Q ≤ 19) in any read exceeded 50% of the read base number; the repeat sequences were also removed. (6) Variant analysis software (GATK) [11] was used to extract the potential sites of SNP and InDel in the whole genome, compared with the reference database Human_GRCh38_dbSNP141. (7) The variants were firstly confirmed by Sanger sequencing in the genomic DNA of the proband (IV1), and then identified in the DNA of the other subjects (IV2, III4, III5). (8) Primer 5 software was applied to design the primers for PCR amplification and sequencing the gene fragments spanning a *TRIOBP* (NM_001039141.2) variant (*TRIOBP-F*, 5′-CTCACGAAGCACCCAACTGGATAA-3′; *TRIOBP-R*, 5′-GAGGTTCTGGAGGCTCTGGGATTG-3′), a *HOMER2* (NM_199330.2) variant (*HOMER2-F*, 5′-CTGCCTTGTGGTGGTGTGT A-3′; *HOMER2-R*, 5′-CAAACGTT GCTGAGTCTGCC-3′) and a *TMC2* (NM_080751.2) variant (*TMC2-F*, 5′-TGGTTCTTCAGTGGCATCGT-3′; *TMC2-R*, 5′-ACTATCTGGGTAATTGATGT GAGT-3′), respectively.

### Bioinformatics analysis

Single-nucleotide variants (SNVs) and insertions/deletions (indels) were compared with the databases of the 1000 Genomes Project, the Exome Aggregation Consortium database (ExAC), Genome Aggregation Database (gnomAD) and allele frequency in the Gene Mutation Database of Chinese (CNGMD). Pathogenicity of variants was classified according to the guidelines of American College of Medical Genetics and Genomics (ACMG). The novelty of gene variations was evaluated by using the ClinVar software.

## Results

### Analysis of the clinical manifestations

A four-generation family with autosomal recessive hereditary deafness was included in this study. All the family members had normal communication ability, except for the proband and her younger brother who showed serious hearing problems. Pure tone hearing threshold test revealed a severe (61–80 dB SPL) to profound (> 80 dB SPL) symmetric hearing impairment for the two siblings (Fig. 1).

### Identification of the pathogenic variant

**Fig. 2:**
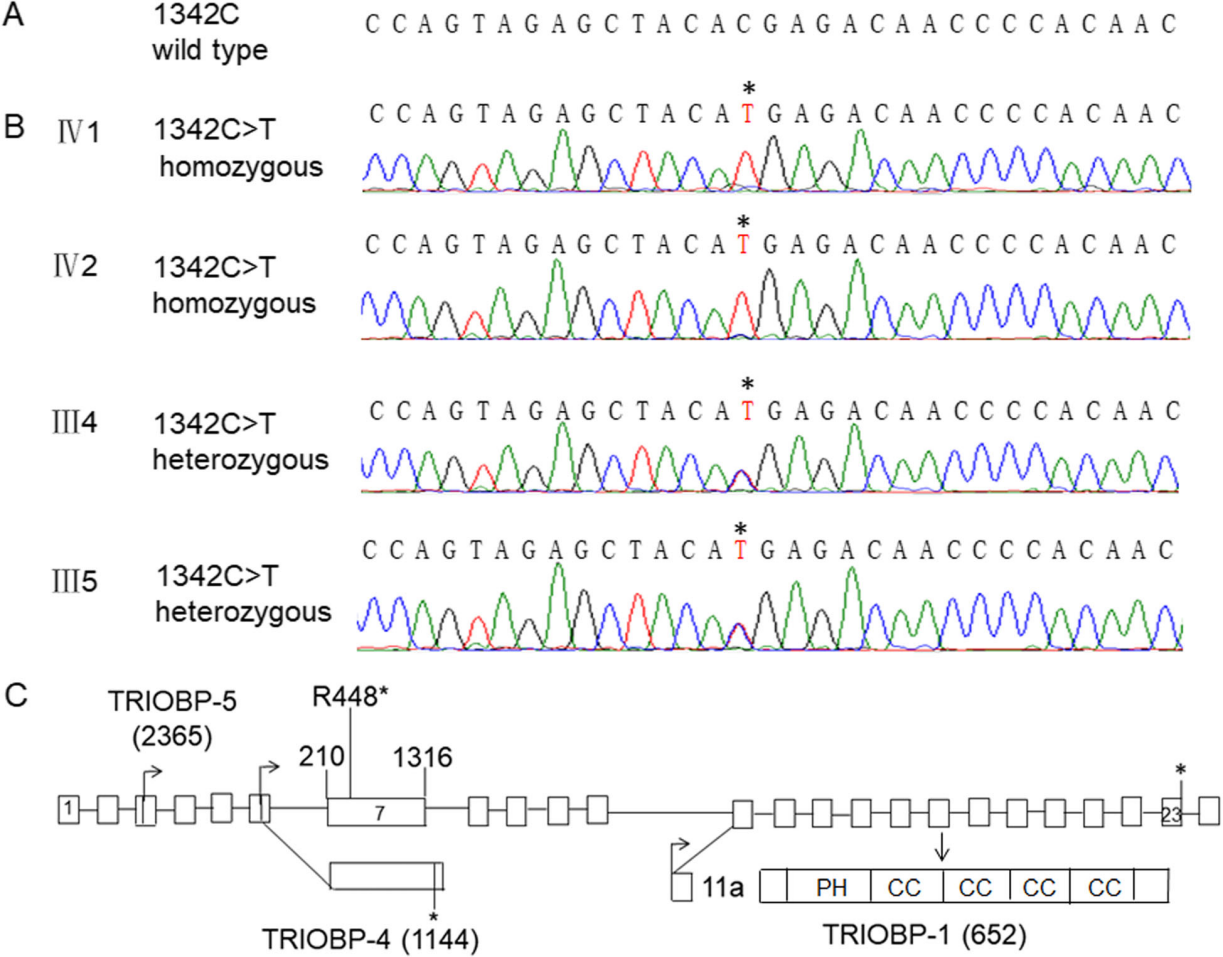
Identification of the pathogenic *TRIOBP* variant in the proband’s family. **a**. Sequence of the wild type *TRIOBP* gene fragment spanning the variant site (1342C). **b**. Identification of the *TRIOBP* variant. The proband (IV1) and her younger brother (IV2) carry a homozygous 1342C > T variant, and her mother (III45) and father (III54) carry a heterozygous 1342C > T variant, as indicated by stars. **c.** Localization of the pathogenic *TRIOBP* variant. Schematic representations of the genomic structure and alternative transcript classes of *TRIOBP: TRIOBP-5*, *TRIOBP-4* and *TRIOBP-1* [1, 5, 7]. The novel c.1342C > T variant is located in exon 7 of *TRIOBP-5* gene and alters the coding codon (Arg448) to a stop codon. Arrows, alternative translation start sites; *alternative stop codon; PH, pleckstrin homology domain; CC, coiled-coil domains

WES was performed on the genomic DNA from the proband. Variant rate less than or equal to 0.01 in CNGMD was used as the frequency cut-offs. A *TRIOBP* c.1342C > T (p.Arg448*) variant with an allele frequency of 0.00002 in ExAC database was further identified. The gnomAD frequency of the variant for SAS is 0.0000 (https://www.ncbi.nlm.nih.gov/snp/rs773152243). The *TRIOBP* c.1342C > T (p.Arg448*) variant is likely pathogenic according to the ACMG guidelines for classification. To verify the results, Sanger sequencing was performed on the DNA of the four family members. It was found that the two siblings carried a homozygous *TRIOBP* c.1342C > T (p.Arg448*) variant, and each of their parents carried a heterozygous *TRIOBP* c.1342C > T (p.Arg448*) variant (Fig. 2). The *TRIOBP* c.1342C > T variant was found to meet the requirement of autosomal recessive disorders, that is, homozygous variant or heterozygous variant in the offspring of parents with a heterozygous variant.

In addition to the *TRIOBP* c.1342C > T variant, a heterozygous *HOMER2* c.826A > G (p.Ile276Val) variant with an AF of 0.002610 and a heterozygous *TMC2* c.834 + 2 T > G variant in the noncoding splicing region were also identified. According to the ACMG standards and guidelines, the *TMC2* and *HOMER2* variants were classified as VUS (variants of uncertain significance). Sanger sequencing results revealed that the subjects of IV1, IV2 and III5 were carriers of a heterozygous *HOMER2* c.826A > G variant and the subjects of IV1 and III4 were carriers of a heterozygous *TMC2* c.834 + 2 T > G variant. The results indicate that the compound heterozygous variants of *HOMER2* c.826A > G and *TMC2* c.834 + 2 T > G in the proband are inherited from her parents.

## Discussion

The cochlear hair cells in the inner ears detect sound by deflection of stereocilia. At the base of the stereocilia, the actin filaments are packed to form rootlets, which are boundled by TRIOBP and extend into the cell body [12, 13]. The rootlets act as pivots for the deflecton of the stereocilia. Mice deficient in TRIOBP-5 result in thin rootlets [14], and mice loss of of TRIOBP-5/− 4 fail to develop rootlets [1, 13]. In the present study, the *TRIOBP* gene of the family members harbors a c.1342C > T (p.Arg448*) variant, which may give rise to a truncated protein and result in disruption of the rootlets of stereocilia. This may be the main reason of hearing loss in the two siblings.

It is known that the R1 motif of TRIOBP-5/− 4 is the major actin-binding domain [6]. There are also iregular actin-binding sites in the CC domains or PH domains in TRIOBP-5/− 1 [5, 15, 16]. In this study, the TRIOBP proteins are predicted to be truncated within the R1 motif due to the mutation. Therefore, the R1 and R2 motifs of TRIOBP-5/− 4, and the PH and CC domains of TRIOBP-5 are predicted to be devoid of function [5], which may impair the actin binding activity of the TRIOBP proteins.

TRIOBP is not only a structure protein, but also a signal molecule. Study has shown that TRIOBP interacts with Trio protein to form complexes that coordinate actin remodeling. The Trio protein is derived from Dbl-homology guanine nucleotide exchange factors (DH-GEFs), which can also serve as transcription factors [3, 17, 18]. Thus, the *TRIOBP* c.1342C > T (p.Arg448*) variant may also impair the DH-GEFs related pathwaws, contributing to the development of deafness in the individuals.

There were reports that variants in *HOMER2* and *TMC1* genes may cause autosomal dominant non-syndromic deafness [19–21]. In addition to the homozygous *TRIOBP* c.1342C > T (p.Arg448*) mutation, a heterozygous *HOMER2* c.826A > G (p.Ile276Val) variant and a heterozygous *TMC2* c.834 + 2 T > G variant were also identified in the genome of the proband in this study. The *TRIOBP* variant is likely pathogenic, and the *HOMER2* and *TMC2* variants are VUS. The Homozygous *TRIOBP* c.1342C > T (p.Arg448*) variant is consistent with the main clinical phenotypes of the patients, whereas the compound heterozygous *HOMER2* c.826A > G (p.Ile276Val) and *TMC2* c.834 + 2 T > G variants may have little clinical significance, as the parents of the proband show normal communication ability.

## Conclusions

In the present study, a novel *TRIOBP* c.1342C > T (p.Arg448*) variant was identified in a Chinese family. The mutation is predicted to produce a truncated TRIOBP-5 and TRIOBP-4, leading to severe to profound congenital deafness. The findings of this study will broaden the spectrum of the pathogenic variants in *TRIOBP* gene. The characteristics of deafness in the family imply that marriage between close relatives should be avoided to prevent genetic disorders.

## Data Availability

The datasets generated and analysed during the current study are available in the [NCBI SRA] repository, [Accession number: PRJNA633183; Web link: https://www.ncbi.nlm.nih.gov/Traces/study/?acc=PRJNA633183].

## Abbreviations

TRIOBP: Trio- and F-actin-binding protein
DFNB28: Deafness of autosomal recessive type 28
WES: Whole exome sequencing
PH: Pleckstrin homology domain
CC: Coiled-coil domains
BERA: Brainstem evoked response audiometry
dB SPL: Decibel sound pressure level
SNVs: Single-nucleotide variants
indels: Insertions/deletions
ExAC: Exome aggregation consortium database
gnomAD: Genome aggregation database
CNGMD: The gene mutation database of Chinese
ACMG: American college of medical genetics and genomics
AF: Alternative allele frequency
VUS: Variant of uncertain significance
DH-GEFs: Dbl-homology guanine nucleotide exchange factors
HOMER2: Homer scaffolding protein 2
TMC1: Transmembrane channel-like 1.
